# Procalcitonin kinetics within the first days of sepsis: relationship with the appropriateness of antibiotic therapy and the outcome

**DOI:** 10.1186/cc7751

**Published:** 2009-03-16

**Authors:** Pierre Emmanuel Charles, Claire Tinel, Saber Barbar, Serge Aho, Sébastien Prin, Jean Marc Doise, Nils Olivier Olsson, Bernard Blettery, Jean Pierre Quenot

**Affiliations:** 1 Service de Réanimation Médicale, Hôpital Le Bocage, C.H.U. de Dijon, 21000 Dijon, France; 2 Service d'Epidémiologie et d'Hygiène Hospitalière, Hôpital Le Bocage, C.H.U. de Dijon, 21000 Dijon, France; 3 Laboratoire d'Immunologie, Hôpital Le Bocage, C.H.U. de Dijon, 21000 Dijon, France

## Abstract

**Introduction:**

Management of the early stage of sepsis is a critical issue. As part of it, infection control including appropriate antibiotic therapy administration should be prompt. However, microbiological findings, if any, are generally obtained late during the course of the disease. The potential interest of procalcitonin (PCT) as a way to assess the clinical efficacy of the empirical antibiotic therapy was addressed in the present study.

**Methods:**

An observational cohort study including 180 patients with documented sepsis was conducted in our 15-bed medical intensive care unit (ICU). Procalcitonin measurement was obtained daily over a 4-day period following the onset of sepsis (day 1 (D1) to D4). The PCT time course was analyzed according to the appropriateness of the first-line empirical antibiotic therapy as well as according to the patient outcome.

**Results:**

Appropriate first-line empirical antibiotic therapy (n = 135) was associated with a significantly greater decrease in PCT between D2 and D3 (ΔPCT D2–D3) (-3.9 (35.9) vs. +5.0 (29.7), respectively; *P *< 0.01). In addition, ΔPCT D2–D3 was found to be an independent predictor of first-line empirical antibiotic therapy appropriateness. In addition, a trend toward a greater rise in PCT between D1 and D2 was observed in patients with inappropriate antibiotics as compared with those with appropriate therapy (+5.2 (47.4) and +1.7 (35.0), respectively; *P *= 0.20). The D1 PCT level failed to predict outcome, but higher levels were measured in the nonsurvivors (n = 51) when compared with the survivors (n = 121) as early as D3 (40.8 (85.7) and 21.3 (41.0), respectively; *P *= 0.04). Moreover, PCT kinetics between D2 and D3 were also found to be significantly different, since a decrease ≥ 30% was expected in the survivors (log-rank test, *P *= 0.04), and was found to be an independent predictor of survival (odds ratio = 2.94; 95% confidence interval 1.22 to 7.09; *P *= 0.02).

**Conclusions:**

In our study in an ICU, appropriateness of the empirical antibiotic therapy and the overall survival were associated with a greater decline in PCT between D2 and D3. Further studies are needed to assess the utility of the daily monitoring of PCT in addition to clinical evaluation during the early management of sepsis.

## Introduction

Bacterial sepsis is a leading cause of morbidity and death among critically ill patients [1-3]. Since the first days of the management of such patients are thought to be critical, both clinical and biological objectives are required to optimize therapies [4-6]. Cumulative evidence supports the fact that severe sepsis arises from the inability of the host to control bacterial growth as well as from an overwhelming inflammatory response that could itself subsequently cause remote organ dysfunction [7]. Eradicating the bacterial invader as well as keeping in check the host's immune response over these so-called golden hours of sepsis are therefore believed to be critical issues. Accordingly, the early administration of appropriate antibiotics leads to a significant improvement in the outcome of the patients with sepsis [8,9]. At least 48 hours, however, are generally required to accurately identify the bacteria, if any, as well as the susceptibility to antimicrobial agents. In addition, the appropriateness of the host response is far more difficult to appreciate routinely.

Elevated levels of serum procalcitonin (PCT), a 116-amino-acid peptide, are strongly associated with systemic bacterial infections [10]. In addition, PCT elevation is thought to be closely dependent on the host cytokine response to microbial challenge, which could be mitigated by the antibacterial effect of antibiotics. Furthermore, the magnitude and time course of this response could be closely related to patient outcome [11,12]. Two studies have emphasized that the relationship between the daily variations of PCT could affect sepsis management regarding the length of antibiotic therapy [13,14]. Little is known, however, about PCT behavior in septic patients according to the appropriateness of the first-line antibiotic therapy. In addition, previously published studies are sparse and provide conflicting results regarding the prognosis value of PCT [15-21].

We therefore conducted an observational study in our 15-bed medical intensive care unit (ICU) to assess to which extent an appropriate empirical antimicrobial therapy could hasten the PCT decrease within the first days of sepsis management.

## Materials and methods

### Study population

Every episode of bacteremia, community-acquired pneumonia and ventilator-associated pneumonia (VAP), as defined below, was prospectively recorded by one of the investigators (PEC) in our ICU throughout the study period, for an epidemiological survey. In addition, PCT dosage was usually performed daily in every patient with suspected sepsis as a reliable tool to improve diagnosis and antimicrobial management [13]. In accordance with French law, no informed consent was required since all measurements were part of routine management. Accordingly, our local Ethics Committee approved the study.

Every patient with either bacteremia, community-acquired pneumonia or VAP, as defined below, on admission to or during the stay in the ICU was therefore eligible for the study if the PCT dosage had been obtained at the onset of clinical sepsis according to the American College of Chest Physicians/Society of Critical Care Medicine Consensus Conference (that is, day 1 (D1)) and at least twice more within next 3 days. No rule was applied regarding the availability of C-reactive protein dosages since our study focused on PCT. Only patients with proven bacterial infection as described below were kept for further analysis, provided they had not received any appropriate antibiotics during the 48 hours prior to the diagnosis of sepsis.

The following information was prospectively collected: the main clinical and epidemiological data at ICU admission, such as age, gender, type of admission (admission was considered surgical in patients who had undergone surgery within the 30 days preceding the onset of bloodstream infection, and medical otherwise), and severity of illness on admission expressed by the Simplified Acute Physiology Score (SAPS) II; patient characteristics at the onset of sepsis and then daily until D4, including main biological results, the septic condition (that is, sepsis, severe sepsis or septic shock), and organ dysfunction expressed by the Sepsis-related Organ Failure Assessment (SOFA) score; the infection source, if known; microbiological findings; and outcome in the ICU (that is, death or discharge).

Other data were collected retrospectively. Each medical chart was therefore reviewed by an external observer (CT), unaware of the purpose of the study, following a standard record sheet. The available PCT measurements were then recorded. Antimicrobial susceptibility testing reports were reviewed by an expert in infectious disease (PEC) unaware of the PCT values as well as of the outcome, in order to determine the appropriateness of the antibiotics administered to the patient as defined below.

### Definitions

One episode of bacteremia was defined as the recovery of any bacterial species, in one or more blood cultures. Patients in whom *Staphylococcus *non-*aureus *were isolated in blood cultures were not eligible, except if at least two consecutive samples grew for the same species harboring the same antibiotic resistance pattern. Blood samples were obtained by blood punctures before being processed using the BACTEC system based both on standard aerobic and anaerobic media coupled with the 9240 automate (Beckton Dickinson Diagnostic Instrument System, Paramus, NJ, USA). Bacteria identification was based on standard methods. The onset of bacteremia was defined as the day when the first positive blood culture was obtained. Two distinct episodes of bloodstream infection were considered in one patient if at least 6 days had elapsed between the two sets of positive blood cultures, provided appropriate therapy was implemented and significant clinical improvement was obtained between the two episodes. This time interval was chosen since previously published data indicate that blood culture negativation is obtained in a median time of around 2 days in patients with bacteremia receiving appropriate antimicrobial treatment.

VAP was considered in every patient submitted to mechanical ventilation for more than 2 days if the following conditions were present: new lung infiltrate on the chest X-ray scan; positive tracheal aspirate cultures (>10^6 ^colony forming units/ml); and Clinical Pulmonary Infection Score > 6 points.

Community-acquired pneumonia was considered in every patient presenting on admission with lung infiltrate on the chest X-ray scan, a history of respiratory symptoms and the presence of a putative lung pathogen within the respiratory secretions and/or a positive urinary antigene for *Streptococcus pneumoniae *or *Legionella pneumophila *serotype 1 using the corresponding Binax assay.

In patients with bacteremia, other septic states were considered according to standard definitions if considered as the infection source (for example, catheter related-bacteremia, urinary tract infection, and so forth).

Sepsis was considered nosocomial if it had appeared more than 2 days after hospital admission.

### Main endpoints

The main clinical endpoint was the appropriateness of the antibiotic therapy given within the first 24 hours following the onset of sepsis (that is, first-line empirical antibiotic therapy). The empirical antibiotic therapy was considered appropriate if the isolated pathogen(s) was (were) susceptible to at least one drug administered at the onset of sepsis according to the corresponding susceptibility testing report. The crude ICU mortality was also considered.

### Measurement of the procalcitonin level

The Kryptor^® ^immunoassay was used according to the manufacturer's instructions (Brahms, Hennigsdorf, Germany). The functional sensitivity of the assay is 0.06 ng/ml. Patients for whom the PCT measurement was either unavailable or were not performed within the 12 hours following the blood sample were excluded from further analysis because of the risk of false-negative results.

### Statistical analysis

Values are expressed as the mean ± standard deviation unless otherwise stated. PCT levels were log-transformed for all analyses. PCT kinetics are expressed as ΔPCT values. ΔPCT was defined as the difference between two subsequent values. For example, ΔPCT D2–D3 was the difference in PCT between the second and third days (ΔPCT D2–D3 = PCT-D3 – PCT-D2) following the onset of sepsis (that is, D1). As a result, ΔPCT D2–D3 > 0 if PCT had increased from D2 to D3. ΔPCT was also expressed as proportions. For example, ΔPCT D2–D3 > 50% meant that PCT has increased by more than 50% between D2 and D3.

Continuous variables were compared with the Mann–Whitney U test. Categorical variables were compared using the chi-square test. We then examined the independent contribution of factors that had been predictive of death in the ICU by univariate analysis. Prior to logistical regression, conformity with the linear gradient of each continuous variable was checked. If the linear model was not appropriate to describe its variations, the variable was transformed according to the parcimonious rule. The candidate variables were then manually entered into a logistical regression model if the associated regression coefficient had *P *< 0.20 by univariate analysis, and then removed if *P *> 0.05 was obtained by multivariate analysis.

It is worth noting that the SAPS II was not entered into the model regardless of the value obtained by univariate analysis. Actually, it has been established that the SAPS II has been validated in a large cohort of patients with various conditions different from sepsis. As a result, although this score is thought to provide a reliable assessment of the mortality risk, it does not specifically measure the risk of death from infectious causes. In addition, since sepsis onset does not always occur on admission, the SAPS II value does not necessarily reflect a patient's condition at this time, especially in terms of organ dysfunction and failure. Actually, sepsis was an ICU-acquired condition in more than one-third of our patients (data not shown). Finally, the sequential measurement of the SAPS II has not yet been validated. The SOFA score was therefore calculated daily during the course of sepsis, and was preferred to the SAPS II as a predictive model of organ dysfunction and outcome. The survival of patients regarding the PCT decrease expressed as proportions were also analyzed through the construction of the corresponding Kaplan–Meier curves compared by the log-rank test.

The relationship between the PCT kinetics and the appropriateness of the first-line antibiotic therapy was investigated through the comparison of the ΔPCT values. A multivariate analysis was conducted following the same rules as described previously.

The diagnosis accuracy of ΔPCT and SOFA for the distinction between survivors and nonsurvivors was then expressed as the area under the corresponding receiver operating characteristic curve.

*P *< 0.05 was considered statistically significant for all analyses. STATA software was used for all analyses (College Station, TX, USA).

## Results

### Patients' characteristics

Between 1 May 2005 and 31 June 2007, 319 patients presented with sepsis on admission to the ICU or during their stay in our ICU. Among these patients, 29 were excluded because the required PCT dosages were not available, 26 were excluded because fungi were isolated, 71 were excluded because bacterial cultures remained sterile and 13 were excluded because appropriate antibiotics had been given within the 48 hours preceding the onset of sepsis. The remaining 180 patients were considered eligible for further analysis.

The main baseline characteristics of the included patients are presented in Table 1. The main source of infection was found to be the lung (51.7%). In more than one-half of the cases of sepsis included, the diagnosis was bacteremia (56.1%). Gram-negative bacteria and Gram-positive bacteria were isolated in the same proportions (48.3% and 43.9% of all isolates, respectively). Gram-negative bacteria of the enterobacteriacae family were the most frequently isolated (32.8% of all isolates). Gram-positive sepsis was mainly caused by *Staphylococcus aureus *and *Streptococcus *spp. (17.2% and 22.2%, respectively). The sepsis was polymicrobial in 7.8% of cases. Septic shock was present in 41.2% of the episodes.

**Table 1:** Baseline characteristics of patients with bacterial sepsis, appropriateness of first-line empirical antibiotic therapy, and outcome

	Overall population (n = 180)	First-line empirical antibiotic therapy		Outcome	
		
		Appropriate (n = 135)	Inappropriate (n = 45)	Survivors (n = 129)	Nonsurvivors (n = 51)
Age (years)	64.0 (15.3)	63.4 (15.9)	65.9 (13.1)	62.2 (15.5)	68.6 (14.0)^a^
Sex (male/female)	122 (67.8%)/58 (32.2%)	87 (64.4%)/48 (35.6%)	35 (77.8%)/10 (22.2%)^b^	91 (70.5%)/38 (29.5%)	31 (60.8%)/20 (39.2%)
SAPS II on admission (points)	46.3 (16.9)	45.2 (17.7)	49.9 (13.7)	41.6 (15.2)	58.1 (15.3)^a^
Time between ICU admission and sepsis (days)	4.7 (8.9)	4.9 (9.7)	4.1 (5.8)	3.8 (8.1)	7.1 (10.3)^a^
Sepsis source					
Pneumonia	93 (51.7%)	68 (54.4%)	25 (55.6%)	59 (45.7%)	34 (66.7%)^a^
Miscellaneous^c^	59 (32.8%)	43 (31.8%)	16 (35.6%)	47 (36.4%)	5 (9.8%)
Urinary tract	28 (15.5%)	24 (17.8%)	4 (8.8%)	23 (17.8%)	12 (23.5%)
Bacteremia	101 (56.1%)	72 (53.3%)	29 (64.4%)	73 (56.6%)	28 (54.9%)
Isolated pathogenes					
Gram-negative	87 (48.3%)	60 (44.5%)	29 (60.0%)^a^	57 (44.2%)	30 (58.8%)^a^
Enterobacteriacae	59 (32.8%)	39 (28.9%)	20 (44.4%)	43 (33.3%)	16 (31.4%)
*Pseudomonas aeruginosa*	16 (8.9%)	12 (8.9%)	4 (8.9%)	7 (5.4%)	9 (17.6%)
Miscellaneous	12 (6.7%)	9 (6.7%)	3 (6.7%)	7 (5.4%)	5 (9.8%)
Gram-positive	79 (43.9%)	65 (51.1%)	14 (31.1%)^a^	62 (48.1%)	17 (33.3%)^a^
*Staphylococcus aureus*	31 (17.2%)	28 (20.7%)	3 (6.7%)	23 (17.8%)	8 (15.7%)
*Streptococcus *spp.	40 (22.2%)	33 (24.4%)	7 (15.5%)	32 (24.8%)	8 (15.7%)
Miscellaneous	8 (4.4%)	4 (3.0%)	4 (8.9%)	7 (5.4%)	1 (1.9%)
Polymicrobial	14 (7.8%)	10 (7.4%)	4 (8.9%)	10 (7.7%)	4 (7.9%)
Sepsis characteristics by D1					
Septic shock	70 (41.2%)	57 (42.2%)	21 (46.7%)	39 (32.8%)	31 (63.3%)^a^
SOFA score (points)	6.2 (3.6)	6.2 (3.5)	6.2 (3.9)	5.4 (3.2)	8.2 (3.5)^a^
Platelet count (giga/l)	208.6 (137.5)	201.4 (123.1)	231.3 (175.2)	229.8 (135.9)	159.2 (129.4)^a^
PaO_2_/FiO_2 _(mmHg)	244 (140)	229.2 (129.7)	285.3 (161.0)^a^	256.3 (140.1)	217.9 (139.2)^b^
Mean arterial pressure (mmHg)	73.5 (19.4)	74.2 (18.7)	71.3 (21.3)	74.4 (18.8)	71.2 (20.7)
Lactate (mmol/l)	3.2 (2.9)	3.4 (3.2)	2.7 (1.8)	2.8 (2.6)	4.1 (3.4)^a^
Bilirubinemia (μmol/l)	28.3 (43.8)	30.8 (47.9)	20.5 (26.1)	28.6 (48.8)	27.7 (28.5)
Creatininemia (μmol/l)	199.1 (181.2)	197.6 (184.2)	203.6 (173.5)	204.7 (196.4)	184.9 (135.9)
C-reactive protein (mg/l)	151.1 (111.9)	159.9 (112.9)	121.1 (105.0)^b^	152.1 (114.2)	148.6 (107.7)
Nosocomial sepsis	98 (54.4%)	70 (51.8%)	28 (62.2%)	64 (49.6%)	34 (66.7%)^b^
ICU length of stay	18.8 (19.9)	19.3 (22.2)	17.5 (13.9)	16.2 (18.2)	25.6 (22.5)^a^

Baseline characteristics for 180 patients with bacterial sepsis, and description of the episodes according to the appropriateness of the first-line empirical antibiotic therapy and the outcome. D1, day sepsis is diagnosed; SAPS II, Simplified Acute Physiology Score II; SOFA, Sepsis-related Organ Failure Assessment; ICU, Intensive Care Unit. ^a^*P *< 0.05. ^b^*P *< 0.20. ^c^Includes soft tissue, central nervous system and catheter-related infections.

### Appropriateness of empirical first-line antibiotic therapy

One-quarter of the patients were given inappropriate antibiotics within the first 24 hours of sepsis management (Table 1). The proportion of Gram-negative bacteria isolated was significantly higher in patients who did not receive appropriate antibiotics than in those who did (60.0% vs. 44.5%, respectively; *P *= 0.04), whereas no difference existed in terms of severity of the disease as assessed by the SAPS II on admission as well as the D1 SOFA score.

Even though the magnitude of the PCT elevation between D1 and D2 seemed larger in patients who were given inappropriate empirical antibiotic therapy than in those who received active molecules, we failed to demonstrate any statistically significant difference (Table 2). In contrast, the PCT variation was significantly different between D2 and D3 (that is, ΔPCT D2–D3) (*P *< 0.01). In addition, the ΔPCT D2–D3 was found to be independently associated with antibiotic appropriateness by logistic regression (Table 3). Finally, PCT elevation by D4 was significantly lower in patients who had received appropriate antibiotics than in those who had not (*P *= 0.03).

**Table 2:** Procalcitonin changes at various time points in patients with bacterial sepsis according to antibiotic therapy

	First-line empirical antibiotic therapy		*P *value
		
	Appropriate	Inappropriate	
PCT at D1 (n = 180; 129 S, 51 NS)^a^	27.2 (62.7)	29.6 (96.7)	0.92
PCT at D2 (n = 163; 117 S, 46 NS)^a^	27.4 (45.1)	40.9 (74.3)	0.09
ΔPCT D1–D2	+1.7 (35.0)	+5.2 (47.4)	0.20
PCT at D3 (n = 164; 117 S, 47 NS)^a^	24.4 (58.4)	34.4 (55.7)	0.12
ΔPCT D2–D3	-3.9 (35.9)	+5.0 (29.7)	<0.01
PCT at D4 (n = 121; 80 S, 41 NS)^a^	17.3 (45.8)	32.4 (46.2)	0.03
ΔPCT D1–D4	-9.1 (46.7)	-0.8 (102.5)	0.01
ΔPCT D3–D4	-8.3 (21.5)	-8.4 (16.6)	0.97

Changes in procalcitonin (PCT) values at various time points in patients with bacterial sepsis according to the appropriateness of the first-line empirical antibiotic therapy. S, survivors; NS, nonsurvivors. ΔPCT D1–D2, procalcitonin decrease between day 2 and day 1 after the onset of sepsis, and so forth. ^a^Missing data are due to insufficient serum sample or death of patients within the 1-day, 2-day or 3-day-period following the onset of sepsis. D1, day sepsis is diagnosed.

**Table 3:** Factors predictive of the appropriateness of first-line empirical antibiotic therapy in patients with bacterial sepsis

	Odds ratio	Variable type	95% confidence interval	*P *value
Gram staining (positive)	2.61	Dichotomous	1.13 to 6.03	0.02
ΔPCT D2–D3	10.29	Continuous	1.66 to 63.9	0.01

Multivariate analysis of factors predictive of the appropriateness of the first-line empirical antibiotic therapy in 147 patients with bacterial sepsis. PCT, procalcitonin; D1, day sepsis is diagnosed; ΔPCT D2–D3, procalcitonin decrease between day 3 and day 2 after the onset of sepsis.

In contrast, no difference was found from D1 to D4 if other potential relevant clinical or biological endpoints were considered (that is, SOFA score, platelet count, blood lactate concentration, mean arterial pressure, PaO_2_/FiO_2_, creatininemia, C-reactive protein), as detailed in Table 4.

**Table 4:** Time course to endpoints other than procalcitonin in bacterial sepsis patients according to antibiotic therapy

	First-line empirical antibiotic therapy		*P *value
		
	Appropriate	Inappropriate	
D1			
SOFA score (points)	6.2 (3.5)	6.4 (4.0)	0.77
Mean arterial pressure (mmHg)	74.2 (18.7)	71.3 (21.3)	0.41
Platelet count (giga/l)	201.4 (123.0)	231.4 (175.2)	0.23
Creatininemia (μmol/l)	197.7 (184.2)	203.6 (173.5)	0.85
Lactate (mmol/l)	3.4 (3.2)	2.8 (1.8)	0.26
PO_2_/FiO_2 _(mmHg)	229 (129)	280 (163)	0.05
C-reactive protein (mg/l)	159.9 (112.9)	121.1 (105.0)	0.11
D2			
SOFA score (points)	6.0 (3.8)	6.1 (4.3)	0.89
Mean arterial pressure (mmHg)	78.6 (18.6)	76.2 (18.7)	0.48
Platelet count (giga/l)	193.0 (128.9)	194.8 (159.9)	0.94
Creatininemia (μmol/l)	183.9 (175.7)	206.0 (185.5)	0.49
Lactate (mmol/l)	2.8 (2.8)	2.1 (0.8)	0.15
PO_2_/FiO_2 _(mmHg)	252 (132)	251 (129)	0.96
C-reactive protein (mg/l)	171.7 (101.8)	159.3 (86.6)	0.59
D3			
SOFA score (points)	5.5 (4.0)	5.7 (4.2)	0.73
Mean arterial pressure (mmHg)	82.3 (19.1)	78.2 (22.3)	0.26
Platelet count (giga/l)	192.1 (127.6)	177.6 (144.2)	0.55
Creatininemia (μmol/l)	173.0 (151.9)	199.9 (189.3)	0.35
Lactate (mmol/l)	2.4 (2.8)	1.8 (0.7)	0.24
PO_2_/FiO_2 _(mmHg)	263 (120.7)	275 (108.6)	0.61
C-reactive protein (mg/l)	176.4 (116.4)	160.0 (86.1)	0.53
D4			
SOFA score (points)	4.5 (3.8)	5.8 (3.9)	0.24
Mean arterial pressure (mmHg)	81.1 (26.8)	77.0 (14.1)	0.53
Platelet count (giga/l)	189.8 (139.3)	135.8 (122.9)	0.14
Creatininemia (μmol/l)	181.1 (161.4)	216.6 (166.7)	0.39
Lactate (mmol/l)	2.6 (3.5)	1.5 (0.5)	0.09
PO_2_/FiO_2 _(mmHg)	249 (122.5)	276 (79)	0.36
C-reactive protein (mg/l)	139.8 (103.7)	122.2 (74.5)	0.48

Time course of relevant endpoints other than procalcitonin in patients with bacterial sepsis according to appropriateness of first-line empirical antibiotic therapy. SOFA, Sepsis-related Organ Failure Assessment; D1, day sepsis is diagnosed.

### Survival analysis

The crude ICU 28 day-mortality was 24.4% in the study population. Age, SAPS II value on admission and SOFA score on the first day of sepsis were found to be associated with an unfavorable outcome (Table 1). Septic shock at the onset of sepsis was also more frequent in nonsurvivors than survivors (63.3% vs. 32.8%, respectively; *P *< 0.01). In addition, these nonsurviving patients were more likely to present with pneumonia and to suffer from Gram-negative infection than were survivors. Among biological and physiological variables, the serum lactates and the platelet count were found to be significantly different between survivors and nonsurvivors.

**Figure 1:**
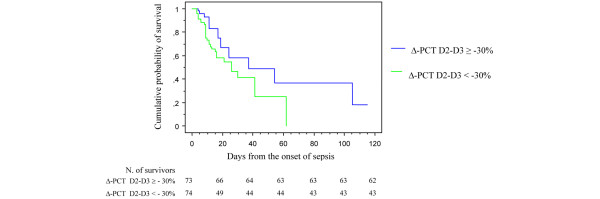
Kaplan–Meier estimated survival after the onset of bacterial sepsis. Kaplan–Meier estimated survival in the intensive care unit after the onset of bacterial sepsis in 147 patients with bacterial sepsis according to the procalcitonin variation between day 3 and day 2 (log-rank test, *P *= 0.04). D1, day sepsis is diagnosed; ΔPCT D2-D3, procalcitonin decrease between day 3 and day 2 after the onset of sepsis.

In contrast, neither the PCT baseline value (that is, the D1 value) nor the D2 value was associated with death in the study population despite a trend toward greater values in the nonsurvivors (Table 5). PCT was found to be significantly higher, however, in nonsurvivors than in survivors by D3 and D4. The ΔPCT D2–D3 value was calculated for only 147 patients because of missing data and because of the death of some patients within this period. ΔPCT D2–D3 was found to be an independent predictor of a bad outcome. In addition, a ΔPCT D2–D3 lower than -30% was associated with death in our study (log-rank test: *P *= 0.04) (Figure 1). ΔPCT D2–D3 was also found to be an independent predictor of a bad outcome in our multivariate analysis (odds ratio = 2.94; 95% confidence interval = 1.22 to 7.09; *P *= 0.02) (Table 6).

**Table 5:** Procalcitonin changes at various time points in patients with bacterial sepsis according to the outcome

	Survivors	Nonsurvivors	*P *value
PCT at D1 (n = 180; 129 S, 51 NS)^a^	21.7 (52.0)	43.0 (107.4)	0.30
PCT at D2 (n = 163; 117 S, 46 NS)^a^	25.7 (41.5)	43.9 (76.3)	0.13
ΔPCT D1–D2	+1.8 (35.9)	+4.8 (44.6)	0.44
PCT at D3 (n = 164; 117 S, 47 NS)^a^	21.3 (41.0)	40.8 (85.7)	0.04
ΔPCT D2–D3	-4.5 (24.0)	+5.4 (52.3)	<0.01
PCT at D4 (n = 121; 80 S, 41 NS)^a^	14.0 (29.1)	34.9 (66.6)	<0.01
ΔPCT D1–D4	-3.2 (38.8)	-14.1 (97.8)	0.05
ΔPCT D3–D4	-5.9 (14.8)	-13.1 (28.2)	0.06

S, survivors; NS, nonsurvivors; PCT, procalcitonin; D1, day sepsis is diagnosed; ΔPCT D1–D2, procalcitonin decrease between day 2 and day 1 after the onset of sepsis, and so forth. ^a^Missing data are due to insufficient serum samples or death of patients within the 1-day, 2-day or 3-day period following the onset of sepsis.

**Table 6:** Multivariate analysis of prognosis factors of outcome in 147 patients with bacterial sepsis

	Odds ratio	Variable type	95% confidence interval	*P *value
Age (years)	1.05	Continuous	1.02 to 1.08	<0.01
SOFA score by day 1	1.28	Continuous	1.12 to 1.45	<0.01
ΔPCT D2–D3 >-30%	2.94	Dichotomous	1.22 to 7.09	0.02
Lung source of infection	3.14	Dichotomous	1.40 to 8.26	0.01

SOFA, Sepsis-related Organ Failure Assessment; D1, day sepsis is diagnosed; ΔPCT D2–D3, procalcitonin decrease between day 3 and day 2 after the onset of sepsis.

**Figure 2:**
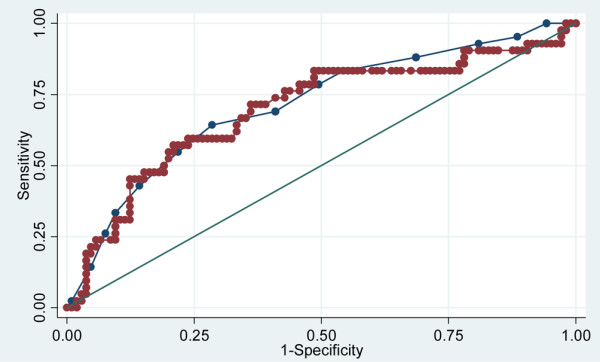
Procalcitonin variation and Sepsis-related Organ Failure Assessment for differentiating between survivors and nonsurvivors. Receiver operating characteristic curves of procalcitonin variation between day 2 and day 3 after the onset of sepsis (red line) and Sepsis-related Organ Failure Assessment (blue line) for differentiating between survivors and nonsurvivors in the intensive care unit in 147 patients with bacterial sepsis. Area under the receiver operating characteristic curve = 0.713 (0.048) and 0.697 (0.051) (mean (standard deviation)), respectively (*P *= 0.80).

**Figure 3:**
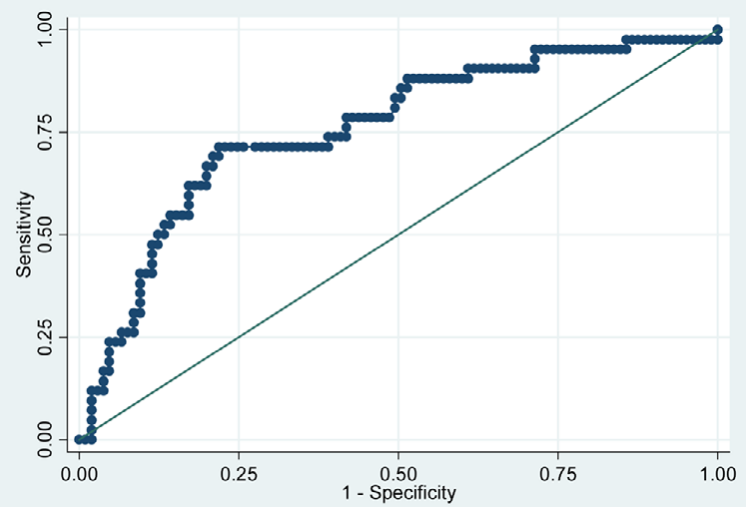
Procalcitonin variation in combination with Sepsis-related Organ Failure Assessment for differentiating between survivors and nonsurvivors. Receiver operating characteristic curves of procalcitonin variation between day 2 and day 3 after the onset of sepsis in combination with Sepsis-related Organ Failure Assessment for differentiating between survivors and nonsurvivors in the intensive care unit in 163 patients with bacterial sepsis. Area under the receiver operating characteristic curve = 0.758 (0.048) (mean (standard deviation)).

The predictive value of ΔPCT D2–D3 was compared with that of the SOFA score on D1 through construction of the corresponding ROC curves. It is worth noting that the areas under the receiver operating characteristic curve achieved by both markers were comparable (mean (standard deviation)) 0.697 (0.051) and 0.713 (0.048), respectively; *P *= not significant) (Figure 2). In addition, we found that the combination of the two led to a significant, although slight, improvement in the predictive value of each factor taken alone (mean (standard deviation)) area under the receiver operating characteristic curve = 0.758 (0.048) (Figure 3).

## Discussion

We show herein that the PCT kinetic within the first 48 hours of management of sepsis could be significantly different according to the appropriateness of the first-line empirical antibiotic therapy. Actually, PCT variations between D2 and D3 were shown to be critical since a significantly greater PCT decline within this period was expected in the patients with appropriate empirical antibiotic therapy. In addition, a trend toward a greater rise in PCT between D1 and D2 was observed in patients with inappropriate antibiotics as compared with those with appropriate therapy. As a result, our findings suggest that patient management might be reassessed if PCT does not decrease by 30% between D2 and D3. In such cases, empirical antibiotic therapy modification towards a broader spectrum should be considered while the microbiological findings, if any, are still pending.

Since the adequacy of early management of critically ill patients with sepsis including antibiotic administration is thought to be critical, objective markers are required. Given the lack of reliability of clinical endpoints such as body temperature, biomarkers are of potential interest. Among them, PCT has appeared as one of the most promising in the setting of severe bacterial sepsis [22]. Only a few studies about the early time-dependent changes of PCT have so far been published, and none of them focused on the appropriateness of the first-line antibiotic therapy. Some experimental data do, however, support the fact that PCT elevation is related to the bacterial load [23]. PCT kinetics during the first days of sepsis could therefore reflect the efficacy of the host immune response with respect to bacterial clearance, with or without the contribution of an appropriate antibiotic therapy. The clinical relevance of such an explanation has already been demonstrated, but only at the late stage of sepsis management (that is, once the continuation of antibiotic therapy becomes a matter of concern) [13,14].

Only one published study provides data about PCT variations according to the adequacy of the empirical antibiotic therapy [24]. In the setting of VAP, these authors failed to demonstrate any difference in either PCT or C-reactive protein variations within the first 5 days of management in patients to whom appropriate treatment was promptly given compared with others. In contrast, a recently published study has shown that a C-reactive protein decline could be more rapidly achieved if empirical antibiotic therapy was effective against the microorganism that was subsequently identified as responsible for the VAP episode [25]. Unfortunately, however, PCT was not measured in that study despite the faster than expected kinetics. As a result, one could argue that the clinical utility of biomarkers is limited since the microbiological findings, if any, are usually available before the fifth day following the onset of sepsis. Our findings, however, suggest that daily monitoring of PCT could be useful to assess the appropriateness of the empirical antibiotic therapy at an earlier stage (that is, within the first 48 hours of management).

Besides these findings, we showed that a decrease of 30% at least was associated with survival. Although low, the predictive value of ΔPCT D2–D3 regarding the outcome was comparable with those of the D1 SOFA score. In contrast, the rise in PCT we generally noticed between D1 and D2 did not appear as a relevant indicator of prognosis. Some authors have reported that the PCT baseline value could differentiate survivors from nonsurvivors in patients with sepsis, while others found that only the level achieved several days later could differentiate the two patient groups [17-19,21]. Differences in the case mix as well as the small size of the study groups could account for such discrepancies. In addition, variations regarding the respective proportions of Gram-negative and Gram-positive bacteria as well as the isolation of yeast in the included patients could offer some additional explanations [26,27]. Previous reports have also pointed out that PCT kinetics, rather than the baseline or the peak values, correlate with patient outcome [28]. Some authors have therefore reported that if PCT remained elevated in critically ill patients with sepsis, then the risk of death was increased, sometimes regardless of the absolute levels [15,16,29].

Although these findings provide a consistent overview of the time course of PCT levels in the patients with sepsis according to the outcome, however, drawing parallels with daily clinical practice remains difficult. Accordingly, changes with time were accurately analyzed in only very few of them. Interestingly, in a study involving 53 patients with septic shock, some authors showed that the rate of PCT decrease (that is, a decrease of 25% at least from baseline value) by D3 was greater in the survivors than in the patients with an unfavorable outcome [20]. Another study investigated the daily kinetics of PCT alone or in combination with other prognosis indicators in 72 patients with septic shock [30]. They found that the combination of an increase in PCT and lactates between D1 and D2 was the best predictor of 28-day mortality, whereas no difference was found when these markers were considered alone. The increase in PCT, however, was not defined in this study.

A study of 75 patients with VAP showed that a decrease in PCT between D5 and baseline (simply defined as a negative Δ value) could predict a good outcome [24]. Similar results were obtained in 100 patients with severe community-acquired pneumonia by comparing D3 PCT levels with baseline values [31]. Unfortunately, PCT was not measured earlier by the authors of the two latter studies. In addition, the LumiTest^® ^(Brahms) was used for PCT measurement in all of these reports, despite lower functional sensitivity when compared with the Kryptor^® ^immunoassay as used in the present study. As a result, PCT variations in patients with low baseline values might be questionable.

It is worth noting that survival was not influenced by the appropriateness of the empirical antibiotic therapy. The fact that the treatment was modified in most patients as early as D2 of sepsis (85.6% of appropriate antibiotic therapy at this time point) may account for this finding. In addition, this could reflect the fact that, in some cases, antibiotic therapy has been modified because of an undesirable course of PCT within this time frame, such as a high ΔPCT D1–D2. Another explanation could be that microbiological findings were available earlier (that is, before D2) in some patients since routine cultures are performed regularly in our ICU. Accordingly, sepsis was more likely to be nosocomial in the patients who were given inappropriate therapy.

Several limitations of our study have to be mentioned. First, the small size of our sample could account for our failure to demonstrate some statistically significant difference between the patients with or without appropriate first-line empirical antibiotic therapy while obvious trends have arisen from our results. This is especially true when considering the difference of ΔPCT D1–D2 between these patients. Second, the present investigation was a single-center study. Any generalization of our data should therefore be cautious. In addition, given the study design, there was a high proportion of missing data. As a result, although excluded patients did not differ from those included in terms of age and severity, one cannot exclude the possibility that PCT values did not follow the same distribution as in the analyzed cohort. Such a weakness is also the strength of our study, however, since PCT dosages were performed in a real-life manner, in theory making it easier to translate our findings to clinical practice. Moreover, we cannot exclude the possibility that some confounding variables have been missed. Hence, although not significant, the higher levels of serum creatininemia could account for the differences between nonsurvivors and survivors with regard to the PCT levels we recorded, as has recently been reported [32].

## Conclusions

Our findings suggest that PCT kinetics within the first 48 hours of sepsis management could be related to the appropriateness of first-line empirical antibiotic therapy as well as to the patient outcome. A prospective study is therefore required to assess the clinical relevance of such results. Actually, the daily variations in PCT in addition to the clinical findings could be used as a surrogate to assess the effectiveness of therapy and to trigger more aggressive therapies and diagnostic investigations in an attempt to improve outcome. As a part of this, broadening the spectrum of the empirical antibiotic therapy should be considered. Accordingly, this hypothesis is currently under investigation through a multicenter prospective interventional study [33].

## Key messages

• The PCT time course within the first days of management of sepsis could be a critical issue in the critically ill patients.

• A marked decreased of PCT between the second and third days might be expected in the patients with appropriate empirical antibiotic therapy and good outcome.

## Abbreviations

ΔPCT: difference between two subsequent values; PaO_2_/FiO_2_: partial arterial pressure of O_2_/fraction of inspired O_2_; PCT: procalcitonin; SAPS: Simplified Acute Physiologic Score; SOFA: Sepsis-related Organ Failure Assessment; VAP: ventilator-associated pneumonia.

## Competing interests

PEC has received payments from Brahms (Hennigsdorf, Germany) to attend several meetings about sepsis management. The other authors declare that they have no competing interests.

## Authors' contributions

PEC designed the study, analyzed the data and drafted the manuscript. SB and CT collected the data and participated in their interpretation. SA performed the statistical analysis. J-PQ, J-MD, SP and BB participated in critical revision of the manuscript. N-OO managed the activity of the Immunology Laboratory.
